# Prognostic value of the CONUT score with immune checkpoint inhibitors as first‐line therapy for metastatic malignant melanoma

**DOI:** 10.1111/1346-8138.17613

**Published:** 2025-02-07

**Authors:** Ken Horisaki, Shusuke Yoshikawa, Shoichiro Mori, Wataru Omata, Arata Tsutsumida, Yoshio Kiyohara

**Affiliations:** ^1^ Department of Dermatology Shizuoka Cancer Center Shizuoka Japan; ^2^ Department of Dermatology Nagoya University Graduate School of Medicine Nagoya Japan

**Keywords:** immune checkpoint inhibitor, irAE, nutrition, skin cancer

## Abstract

The recent availability of immune checkpoint inhibitors (ICIs) has revolutionized the treatment of advanced malignant melanoma (MM). However, many patients with MM do not benefit from ICI treatment. As immunotherapy is associated with significant toxicity and high treatment costs despite its excellent efficacy, it is pertinent to select patients who are likely to respond to ICIs. In this single‐center, retrospective study we investigated whether the controlling nutritional status (CONUT) score is a useful prognostic marker in Japanese patients with advanced‐stage cancer. We analyzed 123 patients with stage IV MM treated with ICIs as first‐line systemic treatment at our hospital between February 2012 and July 2024. Receiver operating characteristic curve analysis was used to calculate the CONUT cut‐off value and CONUT into two groups of ≥3 and ≤2. Progression‐free survival (PFS) and overall survival (OS) were determined using the Kaplan–Meier method, and differences in survival were assessed using the log‐rank test. The Cox proportional hazard regression model was used to evaluate independent prognostic factors. Objective response rate (ORR), PFS, and OS were significantly low in the CONUT ≥3 group, characterized by low nutritional status and high inflammation. Multivariate analysis identified the CONUT score as an independent prognostic factor for both PFS and OS. The CONUT score was not significantly associated with the development of serious immune‐related adverse events. The simplicity of the CONUT score may aid in identifying patients with MM who are suitable candidates for ICI treatment.

## 1 INTRODUCTION

The incidence of malignant melanoma (MM) is increasing, with reports suggesting that up to 20% of patients with MM develop advanced or metastatic melanoma.^1^ Unlike conventional anticancer and molecular‐targeted drugs, immune checkpoint inhibitors (ICIs) target the interactions between cancer and immune cells to enhance immunity against tumors. The recent availability of ICIs, such as anti‐cytotoxic T‐lymphocyte antigen 4 (CTLA‐4) and anti‐programmed cell death 1 (PD‐1)/programmed cell death ligand 1 monoclonal antibodies, has revolutionized the treatment of advanced melanoma. Currently, the most commonly used ICIs for the treatment of advanced melanoma are ipilimumab, an anti‐CTLA‐4 monoclonal antibody, as well as nivolumab and pembrolizumab, which are anti‐PD‐1 antibodies.^2^, ^3^ However, many patients do not benefit from ICI treatments. As immunotherapy is associated with significant toxicity and high treatment costs, despite its excellent efficacy, it is important to select patients who are likely to respond to ICIs. This is particularly important in Asians, who have a higher proportion of acral and mucosal melanoma subtypes and a poorer ICI response than Caucasians.^4^, ^5^ Thus, the discovery and validation of new biomarkers to predict response to ICI treatment is required.

Nutrition and inflammation play important roles in the development and progression of various malignancies. Advanced‐stage malignancies have been shown to present with weight loss and a systemic inflammatory response, which influence cancer cachexia. Therefore, a cancer‐related prognosis has been examined using various scoring systems that reflect nutritional and inflammatory status such as the modified Glasgow Prognostic Score (mGPS), the neutrophil‐lymphocyte ratio (NLR), the systemic immune‐inflammation index (SII), controlling nutritional status (CONUT), the prognostic nutritional index (PNI), and body mass index (BMI).

Focusing on MM and ICIs, many reports indicate that NLR and SII are prognostic factors for ICIs in patients with MM.^6^, ^7^, ^8^, ^9^, ^10^, ^11^, ^12^, ^13^, ^14^, ^15^ Low PNI has also been reported to be a poor prognostic factor in MM if not confined to ICIs;^16^ however, there are no reports on CONUT or mGPS and MM prognosis.

This study aimed to focus on the nutritional and inflammatory status of patients with advanced MM and to seek a convenient way to select groups that are likely to benefit from ICI, particularly among Asian patients. In the present single‐center, retrospective study we investigated whether the CONUT score is a useful prognostic marker in Japanese patients with advanced‐stage MM undergoing ICI as a first‐line systemic treatment.

## 2 METHODS

### 2.1 Study population and data collection

This retrospective cohort study was conducted at Shizuoka Cancer Center in Japan and was approved by the institutional review board (IRB) of our hospital. The requirement for informed consent was waived by the IRB of our hospital because of the retrospective, observational nature of the study. We analyzed patients with stage IV melanoma treated with ICI as first‐line systemic therapy at our hospital between February 2012 and July 2024. The inclusion criteria were as follows: (i) pathologically confirmed melanoma; (ii) primary sites including all skin, mucosa, uvea, and unknown primary sites; (iii) classified as stage IV according to the American Joint Committee on Cancer (AJCC), 8th edition; (iv) treatment with one of the following immune checkpoint inhibitors (ICIs) as first‐line systemic therapy: nivolumab, pembrolizumab, or nivolumab plus ipilimumab combination therapy; and (v) availability of a total lymphocyte count (TL), serum albumin (ALB), and total cholesterol (T‐chol) levels before ICI administration. Clinical data collected included the patient's age, sex, Eastern Cooperative Oncology Group performance status (ECOG‐PS) score, primary tumor location, history of surgery or radiotherapy for the primary site, metastatic organ involvement, *BRAF* mutation status, immune‐related adverse events (irAEs), and blood sampling data such as TL, ALB, T‐chol, and lactate dehydrogenase (LDH) at the start of ICI therapy. Staging followed the AJCC on Cancer 8th edition guidelines, and for unclassified genital, anal, and urinary sites, criteria for cutaneous melanoma were applied. All personal data were managed in strict accordance with the ethical guidelines of the 1964 Declaration of Helsinki.

### 2.2 Definition of the CONUT score

The CONUT score consisted of three factors for each patient: TL, T‐chol, and ALB; the CONUT score was defined as the sum of (1) TL, (2) ALB, and (3) T‐CHOL.^17^ (1) TL ≥1600, 1200–1599, 800–1199, and <800/μL were defined as 0, 1, 2, and 3 points, respectively. (2) ALB ≥3.5, 3.0–3.49, 2.5–2.99, and <2.5 g/dL were scored 0, 2, 4, and 6 points, respectively. (3) T‐CHOL ≥180, 140–179, 100–139, and <100 mg/dL were scored 0, 1, 2, and 3 points, respectively (Table 1).

**TABLE 1:** Definition of CONUT score.

Alb (g/dL)	≥3.5	3.0–3.49	2.50–2.99	<2.5
Alb score	0	2	4	6
TL (/μL)	≥1600	1200–1599	800–1199	<800
TL score	0	1	2	3
T‐chol	180	140–179	100–139	<100
T‐chol score	0	1	2	3

Abbreviations: ALB, serum albumin; T‐chol, total cholesterol level; TL, total lymphocyte count.

### 2.3 Efficacy assessment

The primary outcomes included objective response rate (ORR), progression‐free survival (PFS), and overall survival (OS). Treatment response was assessed using the Response Evaluation Criteria in Solid Tumors, version 1.1. ORR was defined as the proportion of patients who achieved either a complete response (CR) or partial response (PR).

### 2.4 Statistical analysis

Baseline characteristics were compared using the Mann–Whitney *U* test for continuous variables and the chi‐squared test or Fisher's exact test for categorical variables. The chi‐squared test or Fisher's exact test, were used to analyze the ORR. We applied receiver operating characteristic (ROC) curve analysis to calculate the CONUT cut‐off value. PFS and OS were determined using the Kaplan–Meier method, and differences in survival were assessed using the log‐rank test. PFS and OS were determined from the first cycle of ICI treatment. The Cox proportional hazard regression model was used to evaluate independent prognostic factors. Hazard ratios (HRs) with 95% confidence intervals (CIs) were estimated using multivariate Cox proportional hazard regression models. Subgroup analyses of OS according to primary site and treatment regimen were conducted in the same manner. Statistical significance was set at *p* < 0.05. All analyses were performed using EZR software, version 1.55 for Windows XP‐11.

## 3 RESULTS

### 3.1 Cut‐off value according to the ROC curve

**FIGURE 1:**
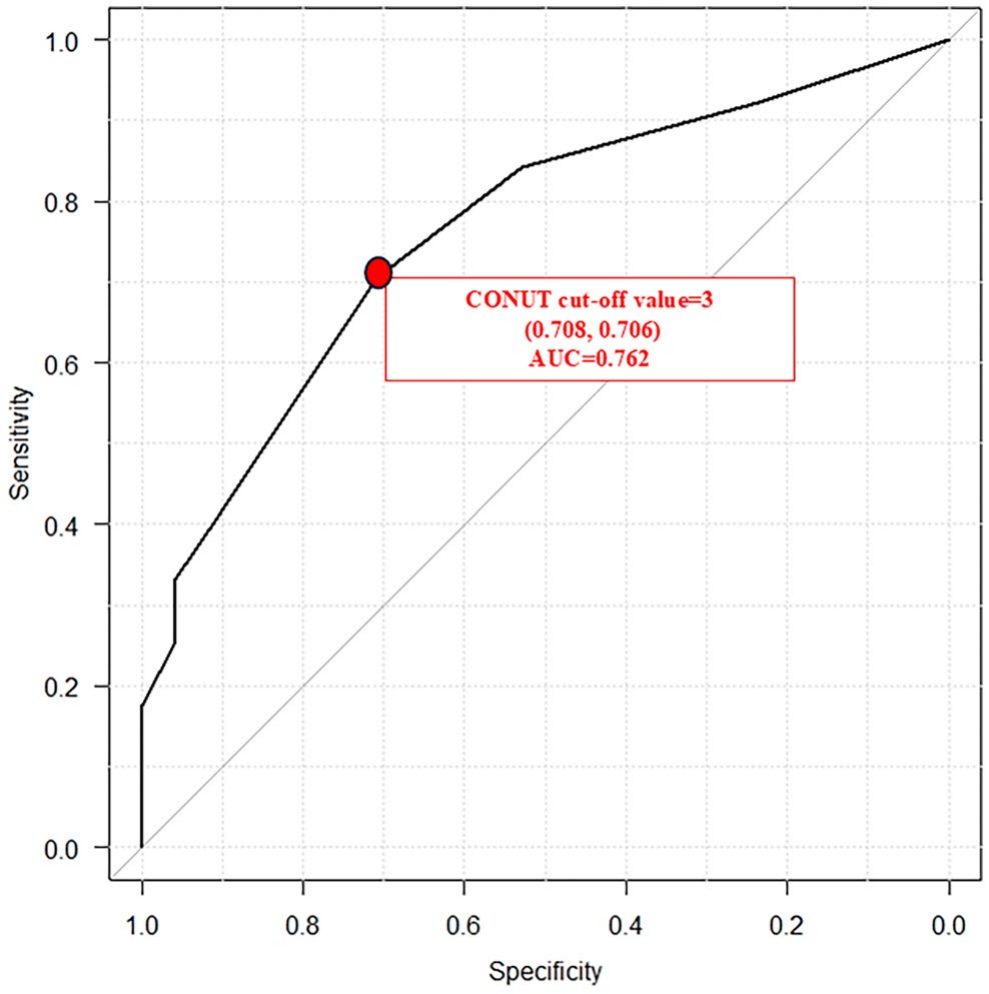
Receiver operating curve analysis. Area under the curve (AUC) on the sensitivity and specificity of the controlling nutritional status (CONUT) score.

The most sensitive and specific cut‐off value for CONUT was determined using ROC curves (Figure 1). The closest point from the top‐left point of the ROC curve was regarded as the optimal cut‐off value, and the cut‐off value for CONUT was 3. The area under the curve (AUC) was 0.762.

### 3.2 Baseline characteristics

The clinical and demographic data of the 123 patients with MM included in this study are shown in Table 2. Sixty‐seven (55.5%) patients had a CONUT score of ≤2 and 56 (45.5%) patients had CONUT score of ≥3. The median follow‐up was 20.2 months in the CONUT ≤2 group and 5.9 months in the CONUT ≥3 group. The CONUT ≤2 group tended to be significantly younger than the CONUT ≥3 group (65.0 vs 70.5 years, *p* = 0.016). About half of both groups were male (56.7% vs 53.6%, *p* = 0.856). There were more patients with ECOG‐PS ≥2 in the CONUT ≥3 group than in the CONUT ≤2 group (16.1% vs 3.0%). There were no significant differences between the CONUT ≤2 and CONUT ≥3 groups except for age and ECOG‐PS. Overall, the primary site was the skin in 62 (50.4%) patients, the mucosa in 43 (35.0%) patients, the uvea in nine (7.3%), and unknown in nine (7.3%) patients. Within stage IV, six (4.9%) patients had M1a, 22 (17.9%) had M1b, 86 (69.9%) had M1c, and nine (7.3%) had M1d. Approximately half of the patients (*n* = 61; 49.6%) had elevated LDH levels. Regarding *BRAF* mutations, 92 (74.8%) patients were tested and 15 were positive for mutations. Regarding treatment at the primary site, 84 patients (68.3%) underwent surgical treatment, while 26 (21.1%) underwent radiotherapy. The first‐line treatment was anti‐PD‐1 antibody monotherapy (PD‐1) in 95 (77.2%) patients and nivolumab plus ipilimumab (NIVO+IPI) in 28 (22.8%) patients. Grade 3 or higher irAEs were observed in 23 patients (18.7%) across all treatment regimens.

**TABLE 2:** Baseline characteristics of patients with malignant melanoma.

Characteristic	Patients group *n* (%)			*p*‐value*
	Total	CONUT ≤2	CONUT ≥3	
Patients *n* (%)	123 (100)	67 (55.5)	56 (45.5)	
Age (years)				
Median [range]	67.0 [28.0, 87.0]	65.0 [28.0, 87.0]	70.5 [31.0, 86.0]	**0.016**
Sex, *n* (%)				
Male	68 (55.3)	38 (56.7)	30 (53.6)	0.856
Female	55 (44.7)	29 (43.3)	26 (46.4)	
ECOG‐PS score, *n* (%)				
0–1	112 (91.1)	65 (97.0)	47 (83.9)	**0.022**
≧2	11 (8.9)	2 (3.0)	9 (16.1)	
Primary site, *n* (%)				
Cutaneous	62 (50.4)	34 (50.7)	28 (50.0)	0.592
Mucosal	43 (35.0)	24 (35.8)	19 (33.9)	
Uveal	9 (7.3)	6 (9.0)	3 (5.4)	
Unknown	9 (7.3)	3 (4.5)	6 (10.7)	
Stage, *n* (%)				
M1a	6 (4.9)	3 (4.5)	3 (5.4)	0.406
M1b	22 (17.9)	15 (22.4)	7 (12.5)	
M1c	86 (69.9)	43 (64.2)	43 (76.8)	
M1d	9 (7.3)	6 (9.0)	3 (5.4)	
LDH value, *n* (%)				
<ULN	62 (50.4)	37 (55.2)	25 (44.6)	0.280
≥ULN	61 (49.6)	30 (44.8)	31 (55.4)	
*BRAF, n* (%)				
Mutant	15 (12.2)	11 (16.4)	4 (7.1)	0.295
Wild	77 (62.6)	39 (58.2)	38 (67.9)	
Not investigated	31 (25.2)	17 (25.4)	14 (25.0)	
Surgery for primary site, *n* (%)				
Yes	84 (68.3)	50 (74.6)	34 (60.7)	0.121
Adjuvant treatment, *n* (%)				
Yes	31 (25.2)	21 (31.3)	10 (17.9)	0.099
ICI for first‐line treatment, *n* (%)				
Anti‐PD‐1 antibody monotherapy	95 (77.2)	52 (77.6)	43 (76.8)	1.000
Nivolumab + Ipilimumab	28 (22.8)	15 (22.4)	13 (23.2)	
irAE grade ≧3, *n* (%)				
No	100 (81.3)	53 (79.1)	47 (83.9)	0.643
Yes	23 (18.7)	14 (20.9)	9 (16.1)	
Outcome, *n* (%)				
Dead	90 (73.2)	47 (70.1)	43 (76.8)	0.423
Alive	33 (26.8)	20 (29.9)	13 (23.2)	

Abbreviations: CONUT, controlling nutritional status; ECOG‐PS, Eastern Cooperative Oncology Group Performance Status; ICI, immune checkpoint inhibitor; irAE, immune‐related adverse event; LDH, lactate dehydrogenase; PD‐1, anti‐programmed cell death 1 monotherapy; ULN, upper limit of normal.

* *p* < 0.05.

*p*‐values of 0.05 or less are bolded.

### 3.3 Objective response

In this cohort, the overall ORR was 22.0% (4.9% CR, 17.1% PR), and the CONUT ≤2 group had significantly better ORR than the CONUT ≥3 group (30.0% vs 12.5%, *p* = 0.020) (Table 3). When analyzed by treatment, patients who received PD‐1 as first‐line therapy showed the same trend as the overall population (30.7% in CONUT ≤2 vs 12.8% in CONUT ≥3, *p* = 0.05). On the other hand, in patients who received NIVO+IPI as first line treatment, ORR was not significantly different between the two groups (26.7% in CONUT ≤2 vs 7.6% in CONUT ≥3, *p* = 0.333).

**TABLE 3:** Comparison of overall response rates between CONUT ≤2 and CONUT ≥3 groups in patients with malignant melanoma.

	Patients group, *n* (%)			*p*‐value*
	Total *n* = 123	CONUT ≤2 *n* = 67	CONUT ≥3 *n* = 56	
Best overall response				
Complete response	6 (4.9)	4 (6.0)	2 (3.6)	**<0.001**
Partial response	21 (17.1)	16 (23.9)	5 (9.1)	
Stable disease	30 (24.4)	23 (34.3)	7 (12.7)	
Progressive disease	57 (46.3)	23 (34.3)	34 (60.7)	
Not evaluable	9 (7.3)	1 (1.5)	8 (14.5)	
Objective response rate, %	22.0	30.0	12.5	**0.020**

* *p* < 0.05.

*p*‐values of 0.05 or less are bolded.

### 3.4 PFS and OS

**FIGURE 2:**
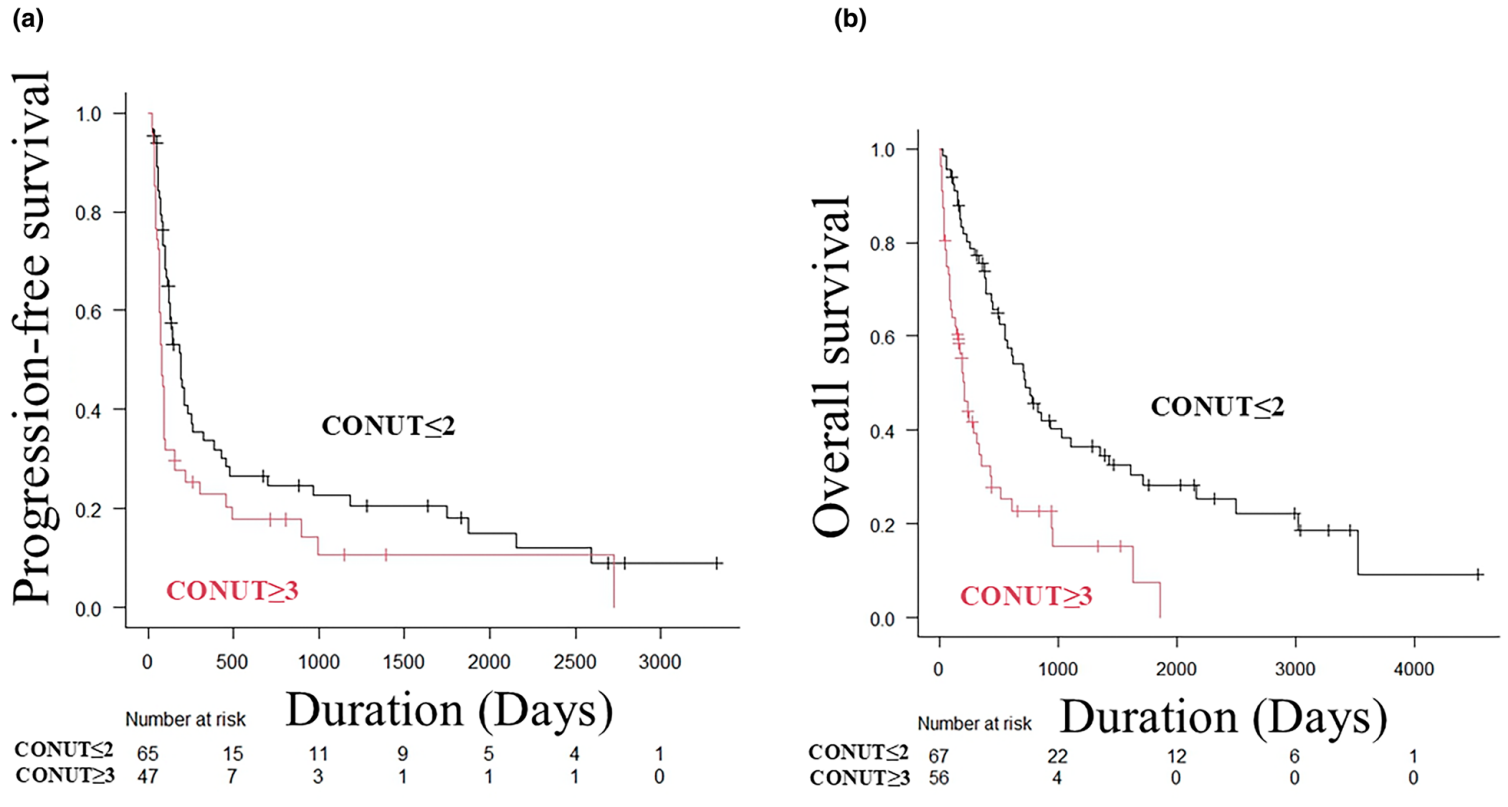
Kaplan–Meier analysis of progression‐free survival (PFS) and overall survival. (a) Kaplan–Meier analysis of PFS in the controlling nutritional status (CONUT) in the CONUT ≤2 and CONUT ≥3 groups. The CONUT ≤2 group had significantly better PFS than the CONUT ≥3 group (median PFS time: 6.2 vs 2.5 months, *p* = 0.017). (b) Kaplan–Meier analysis of overall survival (OS) in the CONUT ≤2 and CONUT ≥3 groups. The CONUT ≤2 group also had significantly better OS than the CONUT ≥3 group (median OS time 23.8 vs 7.0 months, *p* < 0.001).

The CONUT ≤2 group had significantly better PFS than the CONUT ≥3 group (median PFS time: 6.2 months vs 2.5 months, *p* = 0.017) (Figure 2a). With regard to OS, the CONUT ≤2 group also had significantly better OS than the CONUT ≥3 group (median OS time: 23.8 months vs 7.0 months, *p* < 0.001) (Figure 2b). Specifically, the 1‐year PFS was 33.7% vs 22.8% (HR, 1.735; 95% CI 1.103–2.728; *p* = 0.017), and the 3‐year PFS was 22.6% vs 10.6% (HR, 1.696; 95% CI 1.108–2.598; *p* = 0.015) in the CONUT ≤2 and CONUT ≥3 groups, respectively. Additionally, the 1‐year OS was 75.6% vs 32.4% (HR, 4.031; 95% CI 2.22–7.318; *p* < 0.001) and the 3‐year OS was 38.4% vs 15.2% (HR, 2.504; 95% CI1.597–3.927; *p* < 0.001) in the CONUT ≤2 and CONUT ≥3 groups, respectively.

### 3.5 Multivariate analysis for PFS and OS rate

Multivariate analysis was performed to account for potential confounders such as age, ECOG‐PS, and stage (Table 4). For PFS, the CONUT score (CONUT ≥3, HR, 1.65, *p* = 0.030), first‐line systematic therapy (PD‐1, HR, 0.47, *p* = 0.017), and irAE grade ≥3 (experienced irAE grade ≥3, HR, 0.44, *p* = 0.011) were independent predictors of PFS. For OS, CONUT score (CONUT ≥3 HR, 2.68, *p* < 0.001), ECOG‐PS (PS ≥2 HR, 2.76, *p* = 0.008), and primary site (cutaneous HR, 1.65, *p* = 0.042) were independent prognostic factors for OS.

**TABLE 4:** Cox multivariate proportional‐hazards model for progression‐free survival and overall survival in malignant melanoma.

	Progression‐free survival			Overall survival		
	Hazard ratio	95% CI	*p*‐value*	Hazard ratio	95% CI	*p*‐value*
CONUT						
CONUT ≤2	Reference			Reference		
CONUT ≥3	1.65	1.06–2.57	**0.030**	2.68	1.67–4.30	**<0.001**
First‐line systematic therapy						
NIVO+IPI	Reference			Reference		
PD‐1	0.47	0.25–0.87	**0.017**	0.63	0.34–1.17	0.144
Age	1.01	0.99–1.03	0.453	1.01	0.99–1.03	0.516
ECOG‐PS score						
0–1	Reference			Reference		
≥2	1.64	0.57–4.71	0.363	2.76	1.30–5.88	**0.008**
Primary site						
Mucosal	Reference			Reference		
Cutaneous	1.51	0.94–2.45	0.091	1.65	1.02–2.69	**0.042**
Uveal	0.81	0.32–2.07	0.660	1.42	0.59–3.43	0.433
Unknown	1.33	0.56–3.12	0.515	1.38	0.61–3.12	0.438
Stage						
M1a	Reference			Reference		
M1b	0.98	0.32–3.00	0.974	1.81	0.52–6.30	0.352
M1c	1.23	0.43–3.52	0.706	2.03	0.61–6.72	0.250
M1d	1.12	0.31–4.11	0.863	2.54	0.64–10.1	0.184
LDH value						
≥ULN	Reference			Reference		
<ULN	0.85	0.54–1.38	0.493	1.16	0.72–1.87	0.552
irAE grade ≥3						
No	Reference			Reference		
Yes	0.44	0.23–0.83	**0.011**	0.57	0.30–1.10	0.091

Abbreviations: CONUT, controlling nutritional status; ECOG‐PS, Eastern Cooperative Oncology Group Performance Status; irAE, immune‐related adverse event; NIVO+IPI, nivolumab plus ipilimumab therapy; PD‐1, anti‐PD‐1 antibody monotherapy; ULN, upper limit of normal; CI, confidence interval.

* *p* < 0.05.

*p*‐values of 0.05 or less are bolded.

### 3.6 Subgroup analysis of OS by primary site and treatment regimen

**FIGURE 3:**
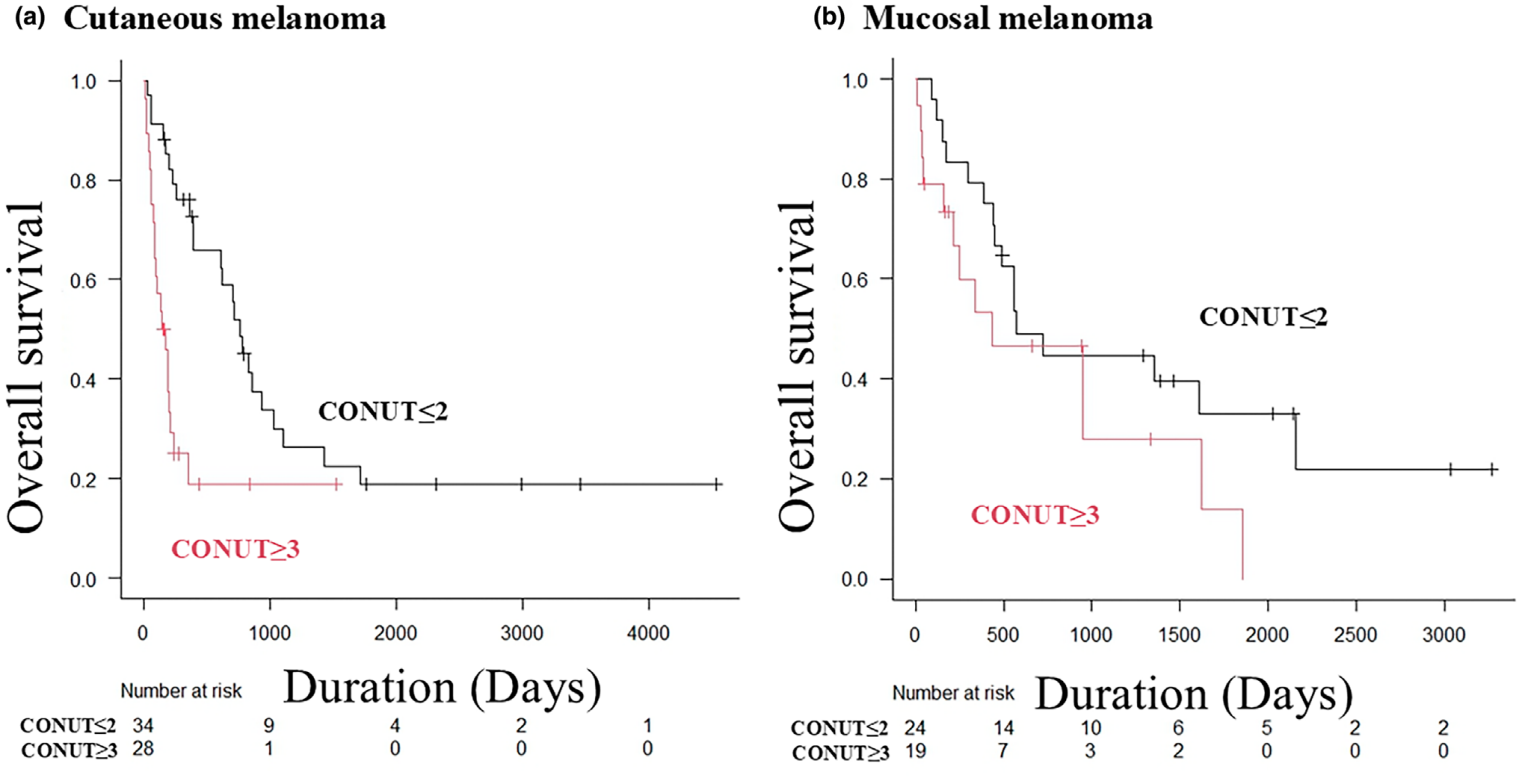
Kaplan–Meier analysis of overall survival (OS) by primary site. (a) Kaplan–Meier analysis of OS in primary cutaneous melanoma. The controlling nutritional status (CONUT) ≤2 group had significantly better PFS than the CONUT ≥3 group (median OS time 25.2 vs 5.2 months, *p* < 0.001). (b) Kaplan–Meier analysis of OS in primary mucosal melanoma. There was no significant difference in OS between the CONUT ≤2 and CONUT ≥3 groups (median OS time 18.9 vs 14.3 months, *p* = 0.13).

**FIGURE 4:**
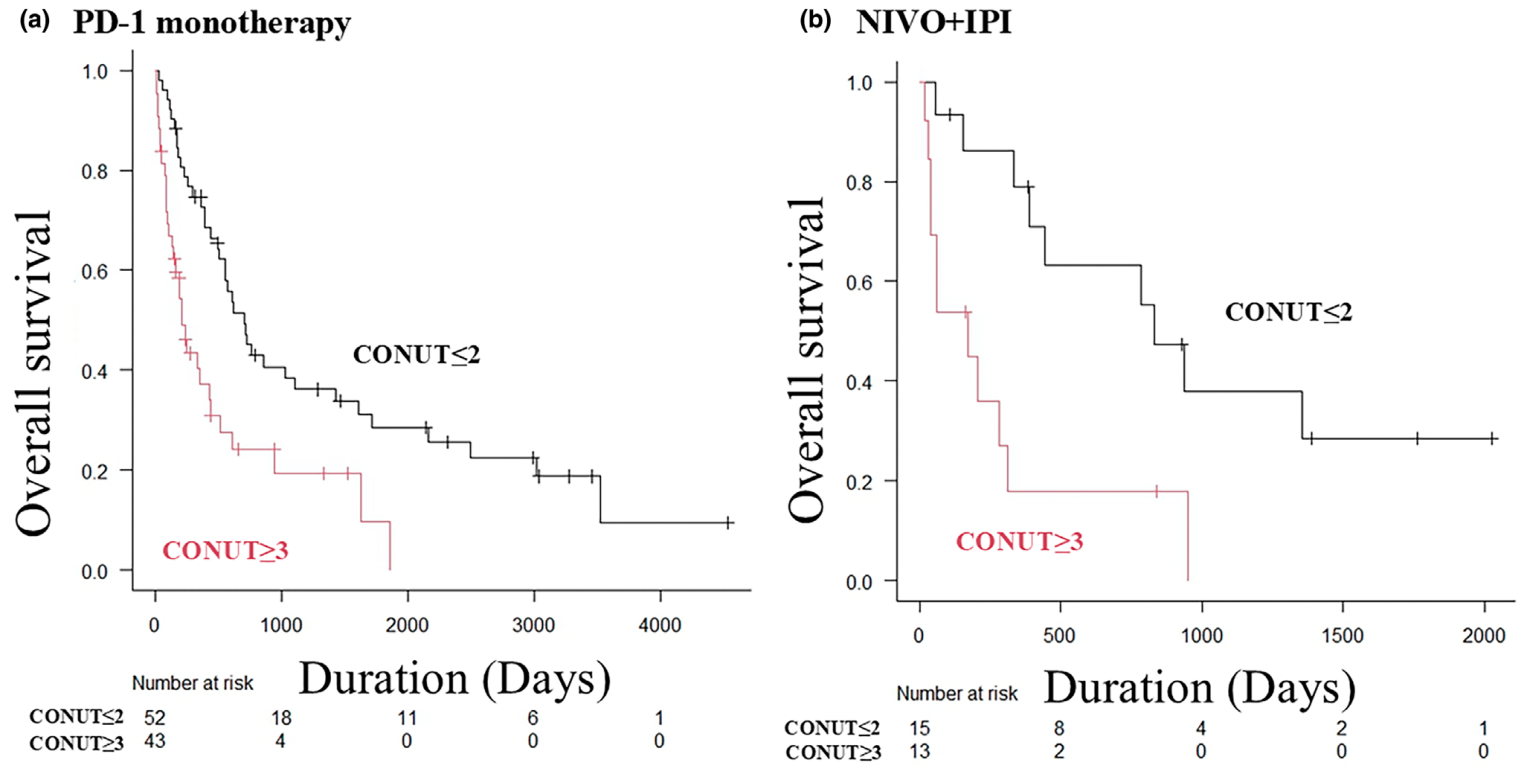
Kaplan–Meier analysis of overall survival (OS) by treatment regimen. (a) Kaplan–Meier analysis of OS in the programmed cell death (PD‐1) group. The controlling nutritional status (CONUT) ≤2 group had significantly better progression free survival than that of the CONUT ≥3 group (median OS time 23.1 vs 7.1 months, *p* < 0.001). (b) Kaplan–Meier analysis of OS in the nivolumab plus ipilimumab (NIVO+IPI) group. The CONUT ≤2 group also had significantly better OS than the CONUT ≥3 group (median OS time 27.3 vs 5.6 months, *p* = 0.005).

As a subgroup analysis by primary site, the OS in primary cutaneous melanomas and primary mucosal melanomas by the CONUT score was examined. Primary cutaneous melanomas exhibited the same pattern as the overall population, with a trend toward poor prognosis in the CONUT ≥3 group (median OS time 25.2 months vs 5.2 months, *p* < 0.001) (Figure 3a). In contrast, primary mucosal melanomas showed no significant trend (median OS time 18.9 months vs 14.3 months, *p* = 0.13) (Figure 3b). Regarding first‐line treatment regimens, the OS in the PD‐1 group and the NIVO+IPI group was also examined. The PD‐1 group experienced significantly worse OS in the CONUT ≥3 group (median OS time 23.1 months vs 7.1 months, *p* < 0.001) (Figure 4a). The NIVO+IPI group also experienced significantly worse OS in the CONUT ≥3 group (median OS time 27.3 months vs 5.6 months, *p* = 0.005) (Figure 4b).

## 4 DISCUSSION

This retrospective, cohort study demonstrated the prognostic utility of nutritional and inflammatory scoring in patients with MM treated with ICI as first‐line systemic treatment. This is the first study to investigate the relationship between the CONUT score and ICI treatment in patients with MM. The main findings of the present study were the following. First, ORR, PFS, and OS were significantly worse in the CONUT ≥3 group; second, CONUT ≥3 was an independent poor prognostic factor in PFS and OS; and third, the CONUT score was not significantly associated with the development of serious irAEs.

Nutritional status and systemic inflammation play important roles in cancer development, cachexia, and therapeutic efficacy.^18^ The CONUT score is calculated using three parameters: ALB, T‐chol, and TL. ALB, T‐chol, and TL indicate protein synthesis capacity, lipid metabolism capacity, and immune function, respectively. Thus, the CONUT score assesses both nutritional and immune status. Many studies have investigated whether NLR and SII, which reflect the nutritional and inflammatory status of patients, are prognostic factors for patients with MM undergoing ICI treatment.^6^, ^7^, ^8^, ^9^, ^10^, ^11^, ^12^, ^13^, ^14^, ^15^ However, while some reported that NLR and SII were prognostic factors,^6^, ^7^, ^8^, ^10^, ^11^, ^13^, ^14^, ^15^ others reported that they were not useful.^9^, ^12^, ^19^ The cut‐off values for NLR and SII also vary from study to study and are not practical in many clinical situations. Therefore, we examined the relationship between the CONUT score and prognosis in patients with MM treated with ICIs to validate another scoring approach. In fact, the CONUT score has been reported to be a prognostic factor in non‐small cell lung cancer (NSCLC) and stomach cancer patients treated with ICIs.^20^, ^21^ In the present study, this score indicated that the CONUT ≥3 group experienced significantly worse ORR, PFS, and OS compared to the CONUT ≤2 group in cases of MM treated with ICIs as first‐line therapy. Additionally, the CONUT score was identified as an independent prognostic predictor of PFS and OS in multivariate analysis. The results indicate that patients with poor nutritional status and high inflammatory conditions benefit less from ICI treatment. In this study, a higher percentage of patients with elevated ECOG‐PS was observed in the CONUT ≥3 group compared to the CONUT ≤2 group (16.1% vs 3.0%, *p* = 0.022), and ECOG‐PS was also an independent prognostic factor for OS. It has been noted that higher CONUT scores correlate with worsened ECOG‐PS, and these factors are associated.^22^ ECOG‐PS is also recognized as a prognostic factor in patients with malignant tumors undergoing ICI treatment^23^ and serves as a straightforward tool for evaluating the nutritional status in MM. Moreover, a significant correlation exists between the CONUT score and another nutritional measure, the Subjective Global Assessment (SGA), as evidenced by a Kappa index of 0.488 (*p* = 0.034).^24^ However, both ECOG‐PS and SGA have subjective elements that might affect their accuracy. In contrast, the CONUT score provides a simple and objective evaluation of nutritional status. Findings from this study underscore the CONUT score's utility as a biomarker in stage IV MM patients receiving ICI therapy.

It is also known that ICI treatment for MM has varying effects depending on the primary site and treatment regimen. In the present sub‐analysis, OS was significantly worse in the CONUT ≥3 group for primary cutaneous melanoma, PD‐1 treatment, and NIVO+IPI treatment groups. CONUT≥3 was also associated with a worse prognosis in primary mucosal melanoma, although not significantly.

Regarding toxicity, irAEs can occur in almost any organ and can sometimes be fatal because of severe toxicity. The CheckMate‐067^2^ study showed that, in patients with MM, the incidence of adverse events of any grade was 96%, 82%, and 86% in the nivolumab plus ipilimumab combination, nivolumab, and ipilimumab groups, respectively, and the incidence of grade 3 or higher adverse events was 55%, 16%, and 27%, respectively. This underscores the importance for clinicians to identify biomarkers that correlate with the development of irAEs. In this study, however, the CONUT scores were not associated with the development of grade 3 or higher irAEs (Table 2). This aligns with prior research on NLR in melanoma.^25^, ^26^ Nonetheless, patients with NSCLC who have low NLR or SII values with low inflammation and good nutritional status are more likely to experience irAEs.^27^, ^28^, ^29^ Similarly, colorectal cancer patients with low pre‐immunotherapy SII experience irAEs more frequently.^30^ In contrast, renal cell carcinoma and gastric cancer patients with a pre‐treatment NLR <4.3 show a significantly reduced risk of developing grade 3–4 irAEs.^31^ It has also been reported that a low pretreatment NLR in hepatocellular carcinoma is associated with a low incidence of irAEs.^32^ These findings suggest that the relationship between NLR, SII, and irAEs varies by malignancy type. Regarding PNI and irAEs, some studies involving NSCLC patients^33^, ^34^ have demonstrated a significant relationship between high PNI and the development of irAEs, though few such studies exist for other malignancies. No study has reported on the relationship between CONUT score and irAEs across any cancer type. In the present study, there was no association between nutritional and inflammatory status and severe irAE, but one reason for this may be the shorter follow‐up period for the CONUT ≥3 group as patients in the CONUT ≥3 group tend to deteriorate more quickly. Given that irAEs are a major reason for discontinuing melanoma treatment, identifying a biomarker that not only predicts the efficacy but also the adverse events of ICIs is crucial, and warrants further research.

In this study, we explored whether a nutritional and inflammatory scoring system different from NLR and SII could serve as a prognostic predictor in patients with MM undergoing ICI treatment. Identifying a straightforward prognostic marker to forecast the efficacy of ICI therapy in MM, particularly among Asians, is critical. Nevertheless, this study has some limitations. First, its retrospective cohort design may introduce selection bias. Second, it was conducted at a single center with a limited sample size. Third, this study represents the initial derivation cohort examining the relationship between the CONUT score and the prognosis of MM, and additional validation cohorts are pertinent for its future practical application.

In conclusion, our research indicates that the CONUT score may act as a prognostic indicator for stage IV MM treated with ICIs as initial therapy. Also, irAEs grade ≥3 did not correlate with inflammation and nutritional status. The CONUT score is very simple but may be useful in selecting patients with MM who should or should not receive ICI treatment.

## CONFLICT OF INTEREST STATEMENT

None declared.

## ETHICS STATEMENT

This study was reviewed and approved by the Ethics Committee of Shizuoka Cancer Center (approval no. J2024‐86; 2024/08/9).
